# Cancer-associated fibroblasts treated with cisplatin facilitates chemoresistance of lung adenocarcinoma through IL-11/IL-11R/STAT3 signaling pathway

**DOI:** 10.1038/srep38408

**Published:** 2016-12-06

**Authors:** Leilei Tao, Guichun Huang, Rui Wang, Yan Pan, Zhenyue He, Xiaoyuan Chu, Haizhu Song, Longbang Chen

**Affiliations:** 1 Medical Oncology Department of Jinling Hospital, Medical School of Nanjing University, Nanjing, 210002 People’s Republic of China

## Abstract

Cancer-associated fibroblasts (CAF) are recognized as one of the key determinants in the malignant progression of lung adenocarcinoma. And its contributions to chemoresistance acquisition of lung cancer has raised more and more attention. In our study, cancer associated fibroblasts treated with cisplatin conferred chemoresistance to lung cancer cells. Meanwhile, Interleukin-11(IL-11) was significantly up-regulated in the CAF stimulated by cisplatin. As confirmed in lung adenocarcinoma cells *in vivo* and *in vitro*, IL-11 could protect cancer cells from cisplatin-induced apoptosis and thus promote their chemoresistance. Furthermore, it was also observed that IL-11 induced STAT3 phosphorylation and increased anti-apoptotic protein Bcl-2 and Survivin expression in cancer cells. The effect could be abrogated by suppressing STAT3 phosphorylation or silencing IL-11Rα expression in cancer cells. In conclusion, chemotherapy-induced IL-11 upregulation in CAF promotes lung adenocarcinoma cell chemoresistance by activating IL-11R/STAT3 anti-apoptotic signaling pathway.

Lung cancer is the most common cancer and the leading cause of cancer-related death in the world^1^. Almost 80% of lung cancer patients were pathologically diagnosed with non-small cell lung cancer (NSCLC), especially adenocarcinoma^2^. For the patients with advanced stage, chemotherapy may be their only treatment option. Resistance to chemotherapeutics agents of lung adenocarcinoma represents the most important dilemma to improve long-term outcomes of patients with advanced stage^3^. Thus, elucidation of the mechanisms involved in chemoresistance of lung adenocarcinoma cells will be helpful to improve clinical treatment of lung adenocarcinoma patients.

Accumulated evidence have demonstrated that chemoresistance results from both genetic and epigenetic regulation of various critical genes. However, most of the studies have focused directly on cancer cells. Recently, tumor microenvironment in facilitating drug-resistance is getting more and more attention^4^. As a major component of tumor stroma, cancer-associated fibroblasts (CAF) contribute to tumor invasion and metastasis through paracrine factors in different types of cancers such as prostate carcinoma^5^, breast cancer^6^ and colorectal cancer^7^. Multiple research have also indicated that CAF can augment cancer cells’ resistance to chemotherapy agents in breast cancer^8^, pancreatic cancer^9^ and head and neck squamous cell carcinoma^10^. Lung adenocarcinoma is one of the most CAF-rich cancers. Therefore, the mechanism of CAF in chemoresistant acquisition of lung adenocarcinoma is getting increasing attention^11^.

The study was designed to screen the differentially expressed genes in CAF induced by cisplatin. Of all the cytokines, IL-11 exhibited a higher expression level in cisplatin-stimulated CAF. IL-11, a member of IL-6 family, initiates intracellular signaling pathways through binding to its receptor IL-11Rα^12^. IL-11 has been detected as a marker for prognosis in gastric adenocarcinoma^13^, breast cancer^14^ and lung cancer^15^. The correlation between IL-11 and chemoresistance of tumor cells has also aroused our interests.

In the study, we found IL-11 facilitated lung cancer cell chemoresistance via IL-11R/STAT3 signaling pathway which promoted anti-apoptosis protein activation. The results of our study suggested that targeting IL-11or its downstream targets could serve as an effective therapeutic strategy for attenuating chemoresistance of lung adenocarcinoma.

## Materials and Methods

### Cancer-associated fibroblast separation and culture

Fresh Lung cancer samples were collected from patients with pathologically diagnosed lung adenocarcinoma who underwent surgical resection in the Jinling Hospital (Nanjing, China). Written informed consent was obtained from all subjects before collecting the samples. All the methods were carried out in accordance with the institutional guidelines and approved by the Ethical Review Committee of Jinling Hospital, Nanjing, China. Tissue samples were cut into small blocks of approximately 1 to 2mm in diameter, digested with trypsin and 0.5% collagenase and filtered through the cell strainer. The fibroblasts were isolated from the cell suspension following the protocol of Anti-Fibroblast MicroBeads human (Miltenyi Biotec, Germany). The separated cells were cultured in F12K medium (Gibco, LifeTech, USA) contained 10% fetal bovine serum (Gibco, Life Tech, USA) with 100 U/mL penicillin, 100 μg/mL streptomycin. After 2 or 3 passages, CAF were identified by morphology (Supplement Fig. 1A) and by staining for α-Smooth Muscle Actin (α-SMA, Supplement Fig. 1B). CAF were negative for epithelial markers, such as E-cadherin (Supplement Fig. 1B,C).

### Cancer Cell Culture

Human lung adenocarcinoma cell A549 and H1975 cell were purchased from the Cell Bank of Chinese Academy of Medical Science (Shanghai, China) and KeyGen Biotech Company (Jiangsu, China) respectively. The cells were cultured in a humidified atmosphere of 5% CO_2_ at 37 °C.

### Realtime RT-PCR

Total RNA of cells were extracted using Trizol reagent (Invitrogen, Carlsbad, CA). Spectrophotometer (BioPhotometer, Eppendorf, Hamburg, German) was used to measure the concentration of total RNA. RT (reverse transcription) was accomplished by PrimeScript^TM^ RT reagent Kit (Takara, Otsu, Japan) following the manufacturer’s protocol. Real-time RT-PCR was performed to determine the gene expression levels in StepOne System (Applied Biosystems, Life Tech, USA). Relative gene expression was analyzed by the ΔΔCt method based on glyceraldehyde-3-phosphate dehydrogenase (GAPDH) levels, and results were expressed as fold change over different conditions. The primer sequence of genes were showed in Supplement Table 3.

### ELISA

Human IL-11 ELISA kits (Neobioscience, China) were used in accordance with the manufacturer’s instructions manual to quantify concentrations of the culture medium of cells and the supernatant of lung or tumor tissue homogenate.

### Transfection of Lentivirus vector or siRNA interference

Lentivirus vector expressing IL-11(LV-IL-11) and negative control were purchased from Realgene Company (Nanjing, Jiangsu). Lentivirus vector with shRNA targeting IL-11 receptor (LV- IL-11Rα RNAi target 5′-GGACCATACCAAAGGAGAT-3′) and negative control were purchased from Genechem Company (Shanghai, China). CAF were planted into 24-well plates (2 × 10^5^cells/well) and transfected with Lentivirus vector expressing IL-11and negative control followed by the manufacturer’s protocol. Lung adenocarcinoma cells (A549 and H1975) were planted into 24-well plates (2 × 10^5^cells/well) and transfected with Lentivirus vector expressing IL-11R interfering RNA and negative control. Polyprene (Sigma, USA) at the concentration of 10 μg/ml was added to enhance the infection. Puromycin (1 μg/ml, Sigma, USA) were utilized to screen the stably infected cells. The CAF stable infected with LV-IL-11 and negative control were designated CAF-IL-11 and CAF-IL-11 NC respectively. The lung adenocarcinoma cells stably infected with LV-IL-11R interfering RNA were named with A549- IL-11Ri and H1975- IL-11Ri. The controls of them were named A549-IL-11Ri NC and H1975- IL-11Ri NC.

### Western blot

Protein was extracted from the cells(CAF, A549 and H1975 cell) by lysis buffer, separated electrophoretically by 10% SDS-polyacrylamide gel and transferred to a PVDF membrane (Millipore, USA). The membranes were blocked for 2 hours with 5% non-fat milk at room temperature, incubated with first antibody to α-SMA antibody (1:1000, Abcam, USA), E-cadherin (1:1000, Abcam, USA), STAT3 antibody (1:1000, arigobio, China), p-STAT3(Tyr705) antibody (1:1000, Millipore, USA), BCL-2 antibody (1:500, Abcam, USA), Survivin antibody (1:500, Abcam, USA) and GAPDH (1:2000, Abcam, USA) overnight at 4 °C and then incubated with second antibody for two hours at 37 °C. Proteins were then displayed with ECL substrate (Cell Signaling Technology, USA) in accordance with the manufacturer’s instructions. Protein levels were normalized to GAPDH levels.

### MTT assay

Single-lung adenocarcinoma cell suspensions (A549 and H1975) were planted into 96-well plates (2 × 10^3^ cells/well) directly. The cells were cultured in various kinds of conditioned medium (CM) or medium with different concentrations of rhIL-11(R&D Systems, USA) for 24 hours. Cells were treated with different concentrations of cisplatin. Forty-eight hours later, cell survival rate was determined following addition of 0.5 mg/ml MTT solution (Sigma, USA). About four hours later, the medium was replaced with dimethylsulfoxide (DMSO, Sigma, USA) and vortexed for 10 min. Absorbance was measured at 490 nm using a microplate reader (Bio-Rad, USA).

### Colony formation assay

The cells (300 cells/well) were seeded into 6-well plates directly. Then the cells were cultured in different medium according to the experimental protocol. After seven days, cells were fixed with methanol and stained with 0.1% crystal violet. Then the number of visible colonies were counted.

### Flow cytometry analysis

Annexin V-fluorescein isothiocyanate (FITC) apoptosis detection kits (Keygene, China) were used to detect apoptosis rate in accordance with the manufacturer’s instructions. Then early apoptotic rate was measured by flow cytometric analysis following the protocol.

### Animal models

Female BALB/c nude mice (CByJ.Cg-Foxn1nu/J) were purchased from Model Animal Research Center of Nanjing University and maintained under controlled temperature and humidity, and a 12-hours light-dark cycle, with sterile food and water *ad libitum*. All mice were 4–6 weeks old. All the animal experiments were carried out in accordance with the institutional guidelines and approved by the Ethical Review Committee of Comparative Medicine, Jinling Hospital, Nanjing, China. Lung adenocarcinoma cells mixed with CAF were subcutaneously injected into the right flank of the mice. The lung adenocarcinoma cells and CAF were mixed at a ratio of 1:1. Treatment was started when tumor volumes grew to approximately 100–150 mm^3^. The first day of treatment was designated as d1. The dose of cisplatin was 5 mg/kg, intraperitoneal. The cisplatin dose used in mice have been calculated according to the conversion formula of drug concentration between human and animals^16^. Subcutaneous tumor volumes were measured daily by caliper and tumor volumes were calculated by the formula: tumor volume = 0.5 × length × width × width.

### Immunohistochemistry and Immunofluorescence assay

Lung cancer samples were collected from patients before chemotherapy who underwent biopsy or resection in Jinling Hospital (Nanjing, China). All patients enrolled in survival analysis have received cisplatin-based chemotherapy after pathologically diagnosed lung adenocarcinoma. Written informed consent was obtained from all subjects before collecting the samples. All the methods were carried out in accordance with the institutional guidelines and approved by the Ethical Review Committee of Jinling Hospital, Nanjing, China.

Tumor samples from patients and subcutaneous xenotransplanted tumors from mice were fixed in 4% formalin and embedded in paraffin. FFEP were utilized for immunohistochemistry or immunofluorescence staining. Rabbit/mouse anti-human IL-11Rα, a-SMA, PCNA antibodies (Abcam, USA) and Rabbit anti-human IL-11antibody (Novus Biologicals, USA) were used in the experiments. TUNEL staining (Roche, USA) were performed according to the manufacturer’s instructions. The mean percentage of positive cells was assigned to five categories: 0, <5; 1, 5 to 25%; 2, 25 to 50%; 3, 50 to 75% and 4, >75%. The intensity of immunostaining was scored as follows: 1, weak; 2, moderate; and 3, intense. The scores of positive cell percentage and staining intensity were multiplied to produce a weighted score for each case. The positive cell percentage and staining intensity in each specimen were analyzed by Image J (http://imagej.nih.gov/ij/)^17^.

α-SMA is a general myofibroblast marker and can be used to identify CAF. The expression of α-SMA of human lung adenocarcinoma tissue were assessed by using a 5-scale scoring system^18^: 0, almost no detectable CAF; 1, a few spindle-shaped CAF; 2, CAF surrounding cancer cells mainly spindle-shaped, less dense; 3, less dense than grade 4 or CAF not scattered in the entire tumor tissue; and 4, dense.CAF almost distributed throughout the entire tumor tissue. For survival analysis, the α-SMA-stained CAF were grouped into CAF-poor (scores of 0, 1, and 2) and CAF-rich (score of 3, 4) (Supplement Fig. 1G).

For immunofluorescence assay, cells seeded on coverslips which were washed with 1 × PBS and fixed with acetone for 10 minutes on ice. After three washes with 1 × PBS, the cells were incubated with the first antibodies at 4 °C overnight and then followed by one-hour staining with fluorescent secondary antibodies and counterstained with DAPI Fluoromount-G (SouthernBiotech, USA).

### Photograph and Statistical analyses

All images were taken using the inverted fluorescence microscope (Zeiss, Germany) and analyzed with ZEN Light Edition. Data was expressed as mean ± SD. One-way ANOVA analysis of difference was used for comparisons among multiple groups. Student’s *t* tests were used for comparisons between two groups. Survival probabilities were determined by Kaplan–Meier analyses and compared by the log-rank test. SPSS 18.0 software was applied for statistical analysis. p < 0.05 was considered significant.

## Results

### Cancer-associated fibroblasts rich lung adenocarcinoma were more resistant to cisplatin-based chemotherapy

**Figure 1:**
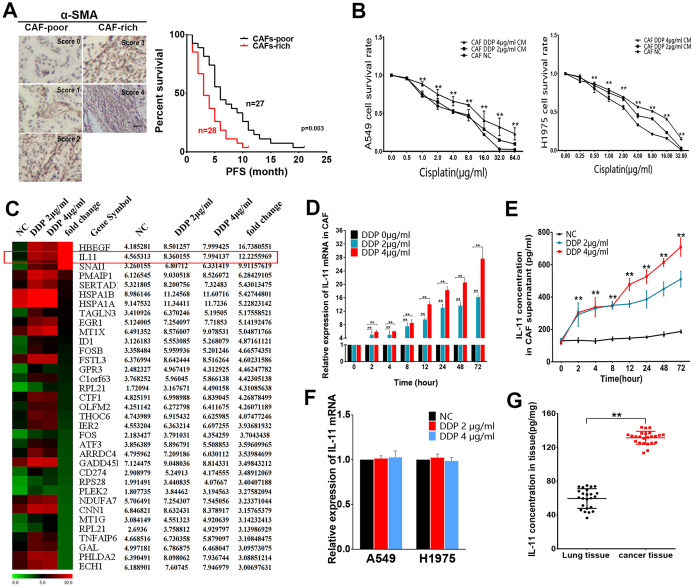
Cancer-associated fibroblasts treated with cisplatin promotes lung cancer cells chemoresistance *in vitro*, meanwhile IL-11 expression was upregulated in CAF. (**A**) The number and distribution of α-SMA positive CAF were scored from 0–4. Representative images are showed. Scale bar, 100 μm. The Kaplan–Meier survival curve demonstrates CAF-rich patients have shorter progression-free survival (PFS) than CAF-poor patients after cisplatin-based chemotherapy (log-rank test, *P* = 0.003). (**B**) A549 and H1975 cells were treated with different concentrations of cisplatin after culture with conditioned mediums from CAF which treated by cisplatin (0 μg/ml, 2 μg/ml, and 4 μg/ml) for twenty-four hours. The cell survival rate was detected by MTT assay.The cells cultured in RPMI 1640 medium were designated as the control. **P < 0.01. (**C**) Different gene expression patterns among CAF treated by different concentration of cisplatin (0 μg/ml, 2 μg/ml, and 4 μg/ml) were assessed using gene array. The CAF treated by cisplatin (0 μg/ml) were as negative control. IL-11 gene is highly expressed cytokine in CAF stimulated by cisplatin compared with normal control. (**D**) IL-11 mRNA levels were upregulated in CAF treated by cisplatin in a dose- and time-dependent manner. **P < 0.01. (**E**) IL-11 protein expression levels in the culture supernatant of CAF stimulated by cisplatin in a dose- and time-dependent manner. **P < 0.01. (**F**) qRT-PCR detecting IL-11 mRNA levels in two lung adenocarcinoma cells treated with cisplatin (0 μg/ml, 2 μg/ml and 4 μg/ml).There is no significant difference between the groups treated with cisplatin and the control group. (**G**) The IL-11 expression levels in normal lung and cancer tissue samples (n = 24) were examined by ELISA. The results showed that the cancer tissue with higher expression level than the normal lung tissue. **P < 0.01. Data are shown as mean ± SD of three replicates and are representative of three independent experiments. **P < 0.01.

To investigate the relationship between CAF and chemoresistance of lung adenocarcinoma patients, a total of 55 clinical tumor tissue samples were collected from lung adenocarcinoma patients who received cisplatin-based chemotherapy. The patients were divided into responding (complete response (CR) + partial response (PR)) and non-responding (stable disease (SD) + progressive disease (PD)) groups according to the Response Evaluation Criteria in Solid Tumors (RECIST). α-SMA is a general myofibroblast marker and can be used to identify CAF. For statistical survival analysis, the α-SMA stained CAF were classified into CAF-poor and CAF-rich groups (Fig. 1A). Statistical analysis showed a correlation between CAF and responses to cisplatin-based chemotherapy in lung adenocarcinoma patients. The CAF-rich patients showed insensitive to cisplatin-based chemotherapy, while the CAF-poor patients exhibited more sensitive to cisplatin-based chemotherapy (P < 0.004, Supplement Table 1). The Kaplan–Meier survival curve demonstrated that the difference of CAF within tumor samples may affect the progression disease-free survival (PFS) of patients. CAF-rich patients have shorter PFS than CAF-poor patients after cisplatin-based chemotherapy (log-rank test, *p* = 0.003) (Fig. 1A). The results indicated that CAF-rich lung adenocarcinoma were more resistant to cisplatin-based chemotherapy.

### Cisplatin-treated CAF induced chemoresistance of lung adenocarcinoma cells *in vitro*

To testified the function of CAF on chemosensitivity of cancer cells, two lung adenocarcinoma cell lines A549 and H1975 were cultured with conditioned medium from CAF treated with cisplatin (DDP 0 μg/ml, 2 μg/ml, and 4 μg/ml) for twenty-four hours (Supplement Fig. 1D). The concentrations of cisplatin treated CAF were calculated from human plasma drug concentration according to the previous paper^19^. The relative sensitivity to cisplatin in lung cancer cells was assessed by MTT assay. CAF-CM (treated by DDP 2 μg/ml and 4 μg/ml) induced resistance to cisplatin in A549 cells compared with the cells cultured with CAF-CM (treated by DDP 0 μg/ml). The cells survival rate of A549 showed no significant difference between the group of CAF-CM treated by DDP 0 μg/ml and the group treated by RPMI 1640 medium. Similar result was also observed in H1975 cells. (Fig. 1B, **p < 0.01).

A transwell co-cultured system of CAF and cancer cells was established to explore the function of CAF inducing chemoresistance of lung adenocarcinoma cells (Supplement Fig. 1E). The pore size of the transwell insert was 0.4 μm. The cells in co-cultured transwell plates were treated with cisplatin (DDP 0 μg/ml, 2 μg/ml, and 4 μg/ml) for twenty-four hours. The survival rates of lung adenocarcinoma cells were measured by MTT assay. The results showed the survival rates of A549 and H1975 were significantly higher when the two cancer cell lines were co-cultured with CAF treated by DDP (2 μg/ml and 4 μg/ml) (Supplement Fig. 1F, **p < 0.01). Taken together, these findings of two co-culture models demonstrated that chemotherapy-treated CAF could induce cisplatin resistance of lung adenocarcinoma cells.

### IL-11 was the key cytokine secreted by CAF after cisplatin treatment

With the evidence from co-culture experiments, we tried to screen the secretory cytokine overexpressed in cisplatin-stimulated CAF by gene array analyses. Of all the significantly up-regulated genes, IL-11 was significantly up-regulated in CAF treated by cisplatin (Fig. 1C). qRT-PCR was applied to verify the results from gene array analyses. The result of qRT-PCR showed that IL-11was up-regulated in CAF treated by cisplatin in a dose- and time-dependent manner (Fig. 1D, **p < 0.01). Furthermore, ELISA assays were used to examine the concentration of secreted IL-11 protein in the supernatant of CAF. The result showed that IL-11protein in the supernatant of CAF was elevated in a cisplatin concentration and time dependent manner (Fig. 1E, **p < 0.01). We detected IL-11 protein levels in CAF supernatant treated with different chemotherapy agents (paclitaxel(PTX), docetaxel(DTX), gemcitabine(GEM) and pemetrexed(PEM)) and radiotherapy (2 Gy and 4 Gy). The results showed that paclitaxel and docetaxel could increase IL-11 expression. The upregulation of IL-11 were not observed in CAF treated by gemcitabine, pemetrexed and radiotherapy. (Supplement Fig. 2C, **p < 0.01).

**Figure 2:**
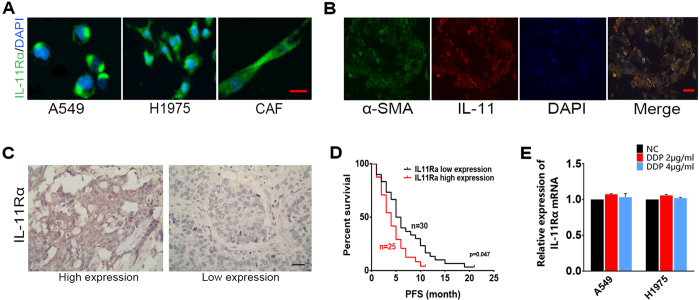
The expression of IL-11Rα in lung adenocarcinoma cells/tissue and its correlation with chemoresistance in lung adenocarcinoma patients. (**A**) The lung adenocarcinoma cells A549, H1975 and CAF were immunostained for IL-11Rα (green), and cell nuclei were stained with DAPI (blue). Only overlay images are shown. Scale bar, 100μm. (**B**) Co-staining of IL-11 (red) with α-SMA (green) in human lung cancer tissue. Cell nuclei were stained with DAPI (blue). Scale bar, 50μm. (**C**) Immunohistochemical staining of IL-11Rα in human lung adenocarcinoma tissue. Representative images are shown. Scale bar, 50 μm. (**D**) The Kaplan–Meier survival curve shows that patients with high IL-11Rα expression have shorter progression-free survival (PFS) than those with low IL-11Rα expression after cisplatin-based chemotherapy (log-rank test, *P* = 0.047 < 0.05). (**E**) The expression of IL-11Rα mRNA of two lung cancer cells treated by cisplatin were analyzed by qRT-PCR.

Furthermore, we detected A549 and H1975 cells could produce IL-11 (Supplement Fig. 2D). However, there was no upregulation of IL-11 mRNA expression in two lung cancer cells after treated with cisplatin for twenty-four hours (Fig. 1F). Similar results were observed in tumor cells. IL-11 mRNA expression was decreased in PBMCs and CD45 + TILs after treated by cisplatin (Supplement Fig. 2E). We also investigated that IL-11 concentration in lung cancer tissue is more abundant than the normal lung tissue (Fig. 1G, **p < 0.01). Moreover, CAF have been validated as major source of IL-11 in tumor stroma by co-staining IL-11 and α-SMA antibodies in human lung cancer tissue section. The results showed that IL-11 were mainly expressed in the CAF stained by α-SMA (Fig. 2B). These findings indicated IL-11 might be a key mediator contributing to maintaining communications between CAF and lung adenocarcinoma cells, which results in chemoresistance.

### Correlation between the expression of IL-11Rα and chemoresistance in lung adenocarcinoma

IL-11 activates the downstream signaling pathways via the IL-11 receptor (IL-11Rα). IL-11Rα expression in lung adenocarcinoma cells and CAF were examined by immunofluorescence and IL-11Rα mainly expressed in cell membrane and cytoplasm of the cells (Fig. 2A). The expression of IL-11Rα in lung adenocarcinoma tissue was stained by immunohistochemistry (Fig. 2C). The statistical analysis demonstrated a correlation between IL-11Rα expression and responses to cisplatin-based chemotherapy in lung adenocarcinoma patients. The patients with high levels of IL-11Rα showed poor response (SD + PD) to cisplatin-based chemotherapy compared with the patients with low levels of IL-11Rα (Supplement Table 2). The Kaplan–Meier survival curve demonstrated that patients with higher levels of IL-11Rα have shorter PFS than the control group after cisplatin-based chemotherapy (log-rank test, *P* = 0.047 < 0.05) (Fig. 2C). The results showed that expression of IL-11Rα was also strongly correlated with chemoresistance of lung adenocarcinoma. However, the IL-11Rα expressions of two lung cancer cells were unchanged after treated by cisplatin (Fig. 2D).

### IL-11 paracrined by CAF induced chemotherapy resistance of lung adenocarcinoma cells

We used lentivirus vector expressing IL-11 to construct CAF-IL-11 cells which stably expressed IL-11. CAF-IL-11 NC cells were served as control. The expression levels of IL-11 of two stably transfected cells were validated by qRT-PCR and ELISA (Supplement Fig. 2A **p < 0.01). To detect the function of CAF-IL-11cells on lung adenocarcinoma cells, conditioned medium of CAF-IL-11 cells and its control cells were collected for further treatment of lung adenocarcinoma cell (A549 and H1975). The sensitivity to cisplatin of A549 and H1975 were assessed by MTT assay. The results of cell survival rate indicated that conditioned medium of CAF-IL-11 could promote cisplatin resistance of lung adenocarcinoma cells (Supplement Fig. 2B, **p < 0.01).

**Figure 3:**
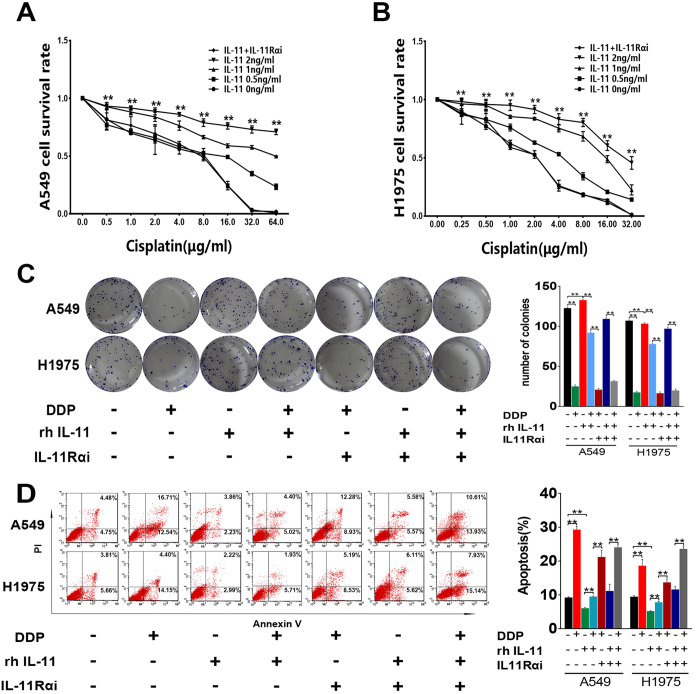
IL-11 contributes to cisplatin resistance, promotes colony formation and decreases cisplatin-induced apoptosis of lung adenocarcinoma cells. (**A,B**) Cell survival rate assay in A549 (**A**) and H1975 (**B**) cells showed that IL-11 contributes LAD cells resistance to cisplatin when rhIL-11 added in the culture medium. Silencing IL-11Rα in LAD cells could neutralize the effect of rhIL-11. **P < 0.01. (**C**) Colony formation assay showing cell proliferation. The histogram represents the number of colonies. **P < 0.01. (**D**) The apoptotic rate in A549 and H1975 cells were analyzed by Flow cytometry. The histogram represents the proportion of apoptotic cells (early and later). Data are shown as mean ± SD of three replicates and are representative of three independent experiments. **P < 0.01.

To further test the effect of IL-11 on lung adenocarcinoma cells, we added recombinant human interleukin-11(rhIL-11) with different concentration to the culture medium of A549 and H1975 cells. After treated with cisplatin, higher cell survival rates of A549 and H1975 cells were observed when cultured in the medium containing different concentrations of rhIL-11. Furthermore, this effect could be neutralized by silencing the expression of IL-11Rα in lung adenocarcinoma cells (A549 and H1975). (Fig. 3A,B, **p < 0.01).

Cisplatin was always used as chemotherapy agent in breast cancer and esophageal cancer. Furthermore, we detected the effect of IL-11 on cisplatin-resistance of breast cancer cells (MBA-MD-231 cells) and esophageal cancer cells (ECA109 cells). The sensitivity to cisplatin of MBA-MD-231 and ECA109 cells were assessed by MTT assay. The results of cell survival rate in ECA109 and MBA-MD-231 cells showed that IL-11 contributes to cells resistance to cisplatin when rhIL-11 was added in the culture medium (Supplement Fig. 2F and G, **p < 0.01, *p < 0.05). These data demonstrated that IL-11 could promote cancer cells resistance to cisplatin treatment.

### IL-11 protects lung cancer cells from cisplatin-induced apoptosis and promotes colony formation

Colony formation assay showed cisplatin could inhibit colony formation capacity of A549 and H1975 cells. However, IL-11 could promote colony formation capacity of lung cancer cells treated with cisplatin. Furthermore, lung cancer cells with IL-11Rα knockdown showed no response to IL-11. (Fig. 3C, **p < 0.01).

DNA damage and subsequent induction of apoptosis may be the primary cytotoxic mechanism of cisplatin. The results of flow cytometry demonstrated that IL-11 markedly decreased the early and later apoptosis rate of lung cancer cells induced by cisplatin. The effect of IL-11 on decreasing apoptosis rate could be neutralized in lung cancer cells with IL-11Rα knockdown (Fig. 3D, **p < 0.01).

### IL-11 paracrined by CAF induced chemotherapy resistance of lung adenocarcinoma *in vivo*

**Figure 4:**
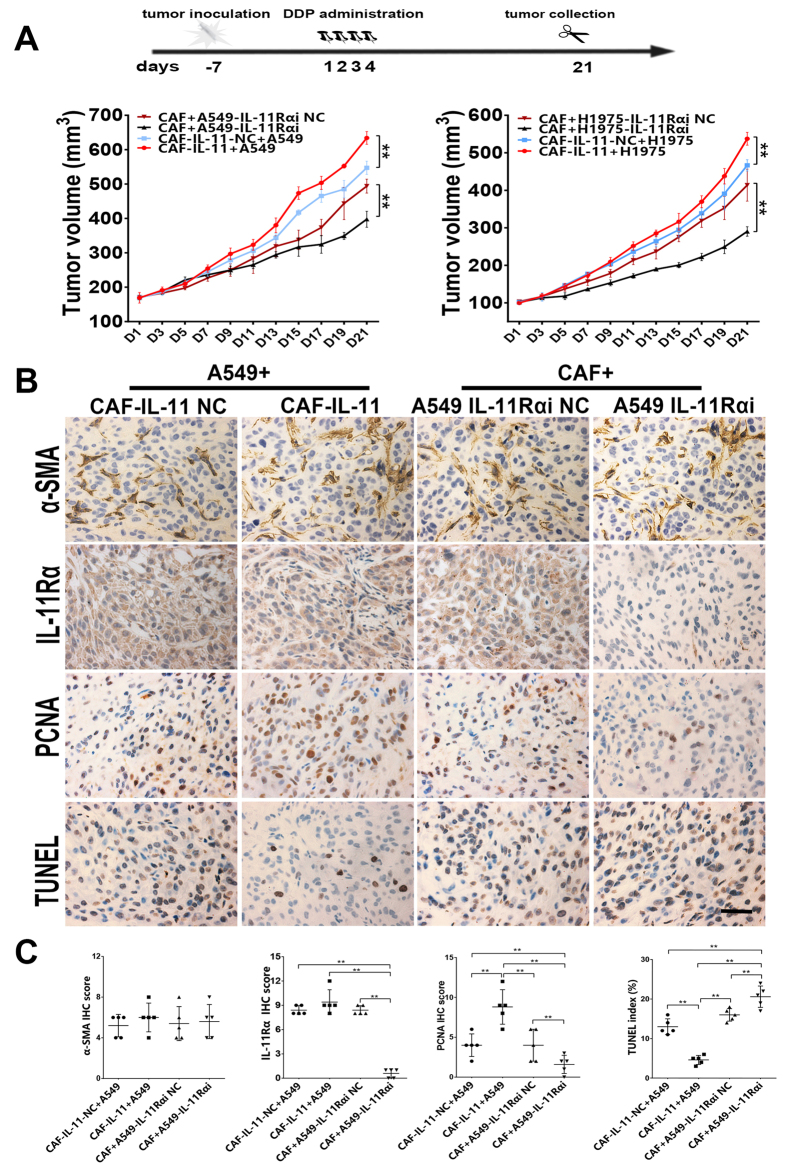
IL-11 paracrined by CAF induced chemotherapy resistance of lung adenocarcinoma*in vivo*. (**A**) Treatment protocol of scheduling administration of cisplatin at a dose of 5 mg/kg in tumor-bearing mice by intraperitoneal injection. Tumor growth curves showed that tumors grew much faster in the group of tumor cells mixed with CAF-IL-11 than other groups. Meanwhile, tumors grew slower in the group of CAF mixed with tumor cells (A549-IL-11Rαi, H1975-IL-11Rαi cells) (n = 5–6, **p < 0.01). **(B)** Representative images of α-SMA-stained, IL-11Rα-stained, PCNA-stained and TUNEL staining in paraffin sections of A549 tumor tissue were shown. Scale bar, 100 μm. Representative images of H1975 tumor tissue were shown in supplement figure. **(C)** The immunohistochemistry semiquantitative scores of biomarkers of were shown. **P < 0.01.

In the xenograft experiments, equal number of CAF and lung adenocarcinoma cells (A549 or H1975) were mixed and implanted subcutaneously in athymic nude mice. Tumor growth curves showed that tumors grew much faster in the group of tumor cells mixed with CAF-IL-11 than other groups. Meanwhile, tumors grew slower in the group of CAF mixed with tumor cells (A549-IL-11Rαi, H1975-IL-11Rαi cells). The results validated that IL-11 secreted from CAF could significantly attenuate the anti-tumor effect of cisplatin. However, tumor growth in the group of CAF mixed with IL-11R knockdown tumor cells was greatly inhibited by cisplatin (Fig. 4A, **p < 0.01).

PCNA (a marker of cell proliferation) and TUNEL assay were used to identify the proliferation ability and apoptosis rate of tumor cells in the xenograft models. The results of TUNEL assay showed that the cell death was reduced in CAF-IL-11-containing tumors. The results of PCNA staining indicated that CAF-paracrine IL-11 could promote tumor cell proliferation (Fig. 4B,C; Supplement Fig. 3A,B, **p < 0.01).

C57BL/6 J mice were supplemented as animal models to observe the possible role of immune system in this study. In the experiment, tumor growth curves showed that tumors grew faster in the group of tumor cells mixed with MF-IL-11 than the other group (Supplement Fig. 3D, **p < 0.01). The results validated that IL-11 secreted from fibroblasts could also attenuate the anti-tumor effect of cisplatin in immune-competent mice.

### IL-11 mediated lung adenocarcinoma cells chemoresistance via IL-11Rα/STAT3 signaling pathway

**Figure 5:**
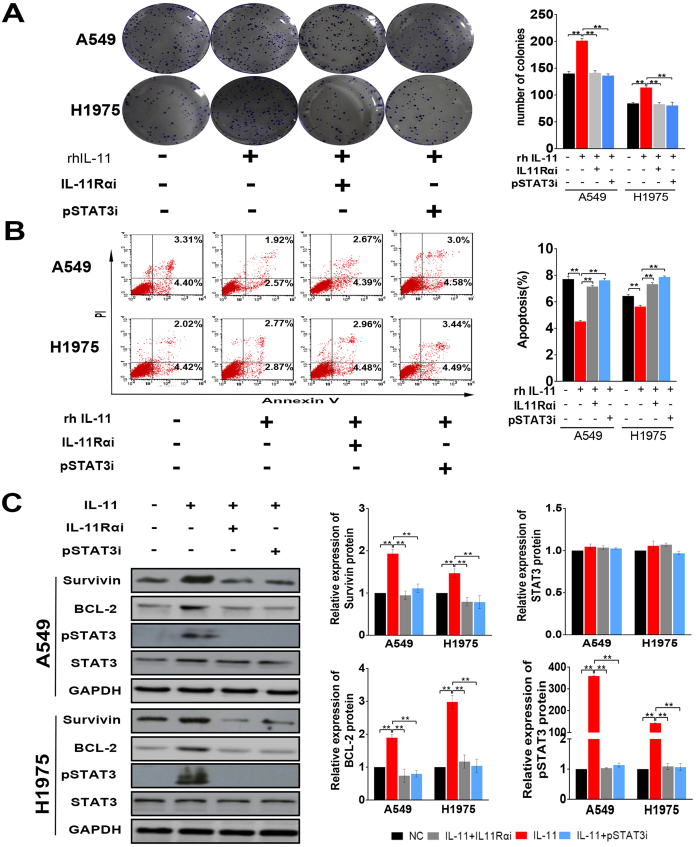
IL-11 enhances the expression of anti-apoptotic proteins by activating STAT3 signaling in binding to IL-11Rα. **(A)** IL-11 promotes the colony formation of lung adenocarcinoma cells.The effect could neutralize through IL-11Rα was silenced or STAT3 phosphorylation were inhibited in cancer cells. The histogram represents the number of colonies. **P < 0.01. (**B**) The apoptotic rate in A549 and H1975 cells were determined by Flow cytometry. The histogram represents the proportion of apoptotic cells (early and later). **P < 0.01. (**C)** Bcl-2, Survivin and p-STAT3 expression in A549 and H1975 cells were detected by Western blot. Glyceraldehyde 3-phosphate dehydrogenase (GAPDH) was used as a loading control. The histogram represents the relative expression of proteins. **P < 0.01. Data are shown as mean ± SD of three replicates and are representative of three independent experiments. **P < 0.01.

Many reports have disclosed that the binding of IL-11 to its receptor IL-11Rα resulted in STAT3 signaling way activation. STAT3 is activated by phosphorylation of its tyrosine and serine residues via signaling from mediators^20^. In the present study, the phosphorylation inhibitor of STAT3 could inhibit the effect of IL-11 on promoting the colony formation and reducing apoptosis rate (early and later) of lung adenocarcinoma cells. (Fig. 5A,B, **P < 0.01)

The expression of crucial proteins in IL-11R/STAT3 signaling way was examined by Western blot. The results showed that IL-11 was capable of inducing STAT3 phosphorylation in A549 and H1975 cells. Furthermore, we examined the expression of the downstream protein of phosphorylated (p)-STAT3. The upregulation of Bcl-2 and Survivin were induced by IL-11 in the lung adenocarcinoma cell. The expression of the proteins (p-STAT3, Bcl-2 and Survivin) showed no obvious changes when the phosphorylation of STAT3 was inhibited or IL11Rα were silenced in lung cancer cells. (Fig. 5C, **P < 0.01).

## Discussion

Chemoresistance has been the most significant impediment of cytotoxic agents in clinical application for advanced NSCLC patients^3^. Studies focusing on chemoresistance of NSCLC might be helpful to improve clinical application of cytotoxic agents. To gain a better insight of the molecular mechanism underlying chemoresistance of NSCLC, lots of studies involving genetic and epigenetic dysregulation of crucial genes have been investigated in our laboratory^21^,^22^,^23^,^24^,^25^,^26^.

Emerging evidence demonstrated that tumor progression relies on crosstalk of cancer cells with fibroblasts, endothelial cells, immune cells and components of the matrix, collectively known as the tumor microenvironment^27^. CAF, as a major component in the tumor microenvironment, have gained increasing attention in tumor microenvironment^28^. The origins of cancer associated fibroblasts within the tumor stroma are ambiguous. CAF are generally defined as a heterogeneous population of cells with multiple sources. Resident fibroblasts are generally regarded as the main derivation for the recruitment of CAF. Other cells, such as mesenchymal cells. epithelial, pericytes, adipocytes and endothelial cells are also origination of CAF^29^.

When cancer cells generate or metastasize to a new organ, cancer cells recruited fibroblasts into tumor masses^28^. The recruitment of CAF is induced by diverse cytokines that secreted by cancer cells and other stromal cells, including transforming growth factor-β (TGFβ), epidermal growth factor (EGF), platelet-derived growth factor (PDGF) and fibroblast growth factor 2 (FGF2), CXCL12 and so on^30^. The tissue type in which the tumor cells grow, the local paracrine environment and the cell type-of-origin are the elements contribute to the recruitment of CAF^31^. All the elements suggested that the relative proportion of CAF may vary in different kinds of tumors.

With the interaction with cancer cells, fibroblasts undergo various morphologic and biological transition. Traditionally, we differentiate CAF in tumor stroma mainly depending on their morphology and some identified biomarkers such as α-SMA and fibroblast activation protein (FAP)^32^. Based on a large amount of research, the recognition of CAF has switched from a negative component to a critical factor accompanying with tumor progression, angiogenesis and metastasis^33^. CAF are able to shape the phenotype of cancer cells through direct cell-to-cell contacts, communicated by secreting soluble factors and modified the components of extracellular matrix^34^. In spite of the effects, little is known about how CAF affect the response of tumor cells to chemotherapy drugs.

In the study, we explored the potential secreted soluble protein or cytokine of CAF in chemoresistance acquisition of lung adenocarcinoma cells. CAF were successfully isolated from lung adenocarcinoma tissue and were identified with the expression of α-SMA. The results of MTT assays confirmed that CAF induced chemoresistance of A549 and H1975 cells in co-cultured condition. To further screen the potential cytokines secreted by CAF that promote chemoresistance of lung adenocarcinoma cells, the differentially expressed genes microarray assay were performed. Among all the upregulated genes, IL-11 is the significantly up-regulated genes in CAF treated by cisplatin.

IL-11, a member of the IL-6 family, activates STAT3 signaling downstream via its receptor IL-11Rα^35^. Many studies have confirmed elevating IL-11 levels in patients with prostate cancer^36^, breast cancer^14^, pancreatic cancer^37^ and lung cancer^38^, are an indicative factor of poor prognosis in these cancers. Secretion of IL-11 by cancer-associated fibroblasts (CAF) promotes cancer cell metastasis in colorectal cancers^39^. Putoczki *et al*. reported IL-11 plays an important role in the progression of inflammation-associated colon and gastric cancers^13^. It has been reported that colorectal and gastric tumor do secret IL-11 which plays an important role in the progression and the differentiation of tumors^40^,^41^. IL-11 was also found to mediate local invasion and distant colonization of cancer cells in hepatocellular carcinoma^42^. The action of IL-11 is primarily determined by the expression of the IL-11Rα. High IL-11Rα chain expression is an independent predictor in renal cell carcinoma^43^. IL-11Rα may also be a target for treatment of endometrial cancer^44^ and prostate cancer^45^. In our study, we did not detect any change of the expression of IL-11Rα in lung cancer cells by cisplatin. The underlying regulation mechanism of IL-11Ra expression, however, remains unclear to this date.

Previous study has been demonstrated that IL-11 may regulate chemoresistance in breast cancer^46^. The present study was designed to discover the mechanism of IL-11secreted from CAF-mediated chemoresistance of lung adenocarcinoma.

Chemoresistance acquisition of cancer cell results from both genetic and epigenetic dysregulation of key genes involving changes in the ABC transporter family, apoptosis, autophagy, cancer stem cell, hypoxia and DNA damage and repair^47^. DNA damage and subsequent induction of apoptosis may be the primary molecular mechanism of cisplatin^48^.

In the study, colony formation and anti-apoptosis experiments showed that IL-11 could enhance anti-apoptosis ability and facilitate colony formation of lung adenocarcinoma cells treated with cisplatin. And the effect could be neutralized by IL11Rα-knockdown in lung adenocarcinoma cells.

**Figure 6:**
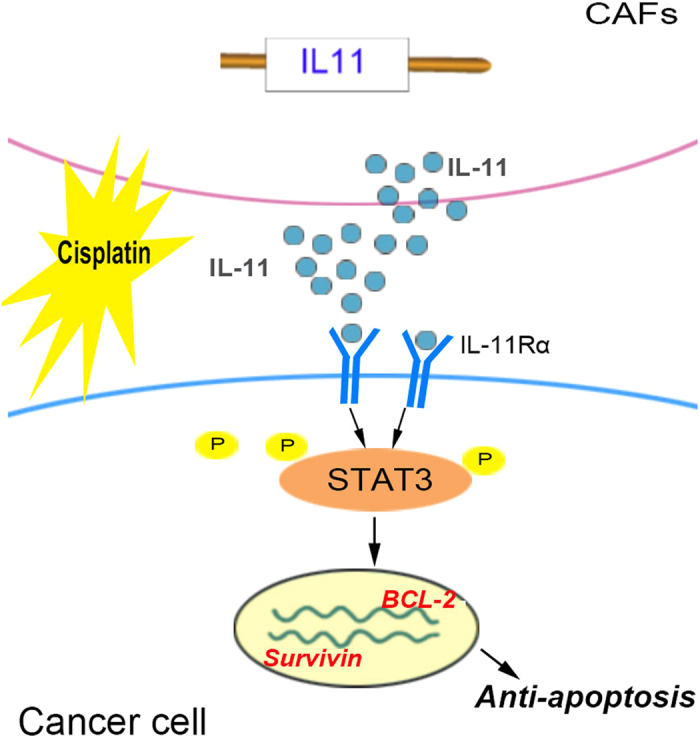
Schematic diagram showing the role of CAF in mediating lung adenocarcinoma cells resistance to cisplatin. IL-11 secreted from CAF induced by cisplatin can activate IL-11Rα/STAT3 signaling pathway in lung adenocarcinoma cells. The phosphorylation of STAT3 could upregulate the expression of anti-apoptotic protein Bcl-2 and Survivin.

Apart from previous reports demonstrating that IL-11 can also activate protein kinase/extracellular signal-regulated kinase (ERK), or the phsophotidylinositol-3 kinase (PI3K) pathways, the STAT3 pathway has been identified as a main pathway activated by chemokines in cancer cells^35^. To better elucidate the mechanisms of chemoresistance induced by IL-11, we found that IL-11 could attenuate cisplatin-induced apoptosis rate of lung adenocarcinoma cells accompanied with the activation of anti-apoptotic STAT3 signaling including Bcl-2 and Survivin overexpression. Bcl-2 was detected to take responsibility for chemoresistance in cancer^49^. Survivin is a member of anti-apoptosis protein and might have a crucial role in conferring chemoresistance to cancer cells^50^. STAT3 has been known to be a critical regulator of apoptosis processes and could upregulate Bcl-2^51^ and Survivin^52^ expression. The enhancement of STAT3 phosphorylation via IL-11 was also observed in lung adenocarcinoma cells. STAT3 phosphorylation inhibitor (Cryptotanshinone, MCE, USA) suppressed STAT3 (Tyr705) phosphorylation and then downregulated Bcl-2 and Survivin expression in lung adenocarcinoma cells. Taken together, our results showed that activation of IL-11/IL-11R/STAT3 anti-apoptotic signaling could augment lung adenocarcinoma cells resistance to cisplatin-induced apoptosis by upregulating Bcl-2 and Survivin (Fig. 6).

However, the mechanism of cisplatin increasing IL-11 expression of CAF is currently not fully understood. Previous reports haven demonstrated that AP-1 could mediate transcriptional activation of the IL-11 promoter^53^,^54^. AP-1 functions as a nuclear regulator in response to extracellular stimuli through the induction of downstream target genes. Cisplatin treatment inducing enhancement of AP-1 expression had been validated in experiment *in vitro*^55^, which may be the one of the possible mechanisms of cisplatin- induced IL-11 expression in CAF.

IL-11 is a thrombopoietic growth factor that directly stimulates megakaryocyte progenitor cells resulting in increased platelet production. Recombinant IL-11 is an efficient cytokine in patients with malignancies who suffered chemotherapy-induced thrombocytopenias. Therefore, the promoting role of IL-11 on chemoresistance of lung adenocarcinoma prompts us to reassess the use of this cytokine in cancer therapy.

In conclusion, cancer associated fibroblasts treated by cisplatin confer chemoresistance to lung adenocarcinoma cells. Meanwhile, IL-11 secreted from CAF could decrease cisplatin-induced apoptosis and promote cancer cells chemoresistance. Furthermore, IL-11 induced STAT3 phosphorylation and increased anti-apoptotic protein Bcl-2 and Survivin expression in cancer cells. The effect could be abrogated by suppressing STAT3 phosphorylation or silencing IL-11Rα in lung adenocarcinoma cells. Therefore, these findings demonstrated IL-11 secreted by CAF after chemotherapy plays a role in cisplatin-based chemoresistance of lung adenocarcinoma patients.

## Additional Information

**How to cite this article**: Tao, L. *et al*. Cancer-associated fibroblasts treated with cisplatin facilitates chemoresistance of lung adenocarcinoma through IL-11/IL-11R/STAT3 signaling pathway. *Sci. Rep.* **6**, 38408; doi: 10.1038/srep38408 (2016).

**Publisher's note:** Springer Nature remains neutral with regard to jurisdictional claims in published maps and institutional affiliations.

## Supplementary Material

Supplementary Files
