# Supplementary material for: Cancer-associated fibroblasts treated with cisplatin facilitates chemoresistance of lung adenocarcinoma through IL-11/IL-11R/STAT3 signaling pathway
Source: Sci Rep. 2016 Dec 6;6:38408. doi: 10.1038/srep38408 (PMC5138853; doi:10.1038/srep38408)

Supplementary files for

Cancer-associated fibroblasts treated with cisplatin facilitates chemoresistance of lung adenocarcinoma through IL-11/IL-11R/STAT3 signaling pathway

Leilei Tao[1*], Guichun Huang[1*], Rui Wang[1], Yan Pan[1], Zhenyue He[1], Xiaoyuan Chu[1], Haizhu Song[1§], Longbang Chen[1§]

[1]Medical Oncology Department of Jinling Hospital, Medical School of Nanjing University, Nanjing, People’s Republic of China, 210002.

*The authors contributed equally to this work.

[§]Corresponding author: Haizhu Song and Longbang Chen.

(email:dr.chenlb@nju.edu.cn)

Supplementary Materials and Methods

Peripheral blood mononuclear cells and CD45+ tumor infiltrating leukocytes separation and culture

Written informed consent was obtained from all subjects before collecting the samples. All the methods were carried out in accordance with the institutional guidelines and approved by the Ethical Review Committee of Jinling Hospital, Nanjing, China.

Human peripheral blood samples were obtained from lung adenocarcinoma patients before chemotherapy. Peripheral blood mononuclear cells (PBMC) were separated from human peripheral blood through density gradient centrifugation using Ficoll-Hypaque (Beijing Chemical Reagents Company, China).

Fresh Lung cancer samples were collected from patients with pathologically diagnosed lung adenocarcinoma who underwent surgical resection in the Jinling Hospital (Nanjing, China). Tissue samples were cut into small blocks of approximately 1 to 2mm in diameter, digested with trypsin and 0.5% collagenase and filtered through the cell strainer. CD45+ tumor infiltrating leukocytes (TILs) were isolated from the cell suspension following the protocol of CD45 MicroBeads human (Miltenyi Biotec, Germany).

The separated cells were suspended in culture medium (RPMI 1640 medium (Gibco, Life Tech, USA) containing 10% fetal bovine serum (Gibco, Life Tech, USA), 100U/mL penicillin and 100μg/L streptomycin) and then cultured in 37C°, 5%CO2. All kinds of the cells were treated with different concentration of cisplatin. Twenty-four hours later, RNA were extracted from cells for further RT-PCR study.

Animal models

C57BL/6J mice were applied as animal models to observe the possible role of immune system in this study. The mice were purchased from Model Animal Research Center of Nanjing University and maintained under controlled temperature and humidity, and a 12-hours light-dark cycle, with sterile food and water ad libitum. All

mice were 4-6 weeks old. All the animal experiments were carried out in accordance with the institutional guidelines and approved by the Ethical Review Committee of Comparative Medicine, Jinling Hospital, Nanjing, China.

Plasmid expressing IL-11 and negative control were purchased from Realgene Company (Nanjing, Jiangsu). Mouse fibroblasts were transfected with plasmid expressing IL-11 and negative control followed by the manufacturer's protocol. The fibroblasts stably infected with plasmid expressing IL-11 and negative control were designated as MF-IL-11 and MF-IL-11 NC respectively. Lewis lung cancer cells mixed with mouse fibroblasts were subcutaneously injected into the right flank of the mice. Treatment was started when tumor volumes grew to approximately150 mm^3. The first day of treatment was designated as D1. The dose of cisplatin was 5 mg/kg, intraperitoneal. The dose of cisplatin used in the mouse was calculated from "Conversion of Animal Doses to Human Equivalent Doses Based on Body Surface Area" in the "Guidance for industry: estimating the maximum safe starting dose in initial clinical trials for therapeutics in adult healthy volunteers". Subcutaneous tumor volumes were measured daily by caliper and tumor volumes were calculated by the formula: tumor volume=0.5×length×width×width.

Supplementary Figure Legends

Figure S1 (A) Morphological features of primary cultured CAF isolated from the primary lung adenocarcinoma tissue. Scale bar, 50μm. **(B)** a-SMA and E-cadherin expression were detected by immunofluorescence, and cell nuclei were stained with DAPI. Only overlay images are shown. Scale bar, 50μm. **(C)** The expression of a-SMA and E-cadherin in CAF isolated from 3 primary LAD tissues was examined by western blot. All the primary cultured CAF expressed a-SMA but did not express E-cadherin, presenting characteristics of CAF. **(D)** Schematic diagram of collection of conditioned medium in the experiment. (**E)** The schematic diagram of co-culture transwell system. **(F)** A549 and H1975 cells were co-cultured with CAF in a transwell co-culture model.

Th cells in the co-cultured system were treated by cisplatin (0μg/ml, 2μg/ml and 4μg/ml) for twenty-four hours. The cell survival rate were detected by MTT assay .***p*<0.01. **(G)** The α-SMA score in tumor stroma. bar, 100μm.

Figure S2 **(A)** The expression of IL-11mRNA and protein of CAF with transfected IL-11 expressing plasmid vector were verified by qRT-PCR and ELISA. ** *P*<0.01. **(B)** A549 and H1975 cells treated with different concentrations of cisplatin and cultured with conditioned medium of CAF with overexpressed IL-11 plasmid and the control. Cell survival rates were measured by MTT assay. ** *P*<0.01. **(C)** IL-11 protein levels in the supernatant of CAF treated by different chemotherapy agents (paclitaxel(PTX), docetaxel(DTX), gemcitabine(GEM) and pemetrexed(PEM)) and radiotherapy(2Gy and 4 Gy). The results showed that paclitaxel and docetaxel could increase IL-11 expression. The upregulation of IL-11 were not observed in CAF treated by gemcitabine, pemetrexed and radiotherapy. ** *P*<0.01. **(D)** IL-11 protein levels of A549 and H1975 lung cancer cells were examined by ELISA. **(E)** qRT-PCR detecting IL-11 mRNA levels in CAF, tumor cells, PBMC and CD45+ TILs. ** *P*<0.01. **(F,G)** Cell survival rate assay in ECA109 and MBA-MD-231 cells showed that IL-11 contributes cells resistance to cisplatin when rhIL-11 added in the culture medium. ** *P*<0.01.

Figure S3 **(A)** Representative images of α-SMA-stained, IL-11Rα-stained, PCNA-stained and TUNEL staining in paraffin sections of H1975 tumor tissue were shown. Scale bar, 100 μm. (**B)** The immunohistochemistry semiquantitative scores of biomarkers of were shown. ** *P*<0.01. **(C)** The concentration of IL-11 protein in xenograft tumor tissues were detected by ELISA. ** *P*<0.01. **(D)** Tumor growth curves showed that tumors grew much faster in the group of Lewis lung cancer cells mixed with MF-IL-11 than the control group. (n=4, ***p*<0.01).

Figure S4 Original westernblots of Figure 5C and Figure S1C. And the westernblots were scanned from the X-ray films. The cropped bands were framed with black lines.

Supplement Table 1. Correlation between α-SMA expression and clinicopathological parameters of lung adenocarcinoma patients

Parameter	α-SMA expression		*p*-value
	CAF-poor(n=27)	CAF-rich(n=28)	
**Age**			0.35
young (< 60 years)	13	10	
old (⩾60 years)	14	18	
**Gender**			0.348
Male	15	19	
Female	12	9	
**Stage**			0.688
Ⅲ	14	13	
Ⅳ	13	15	
**Tumor differentiation**			0.13
Well+moderate	18	13	
poor	9	15	
**Tumor response**			0.004 *
CR+PR	20	10	
SD+PD	7	18	

* $p<0.05$

Supplement Table 2. Correlation between IL-11Rα expression and clinicopathological parameters of lung adenocarcinoma patients

Parameter	IL-11R expression		*p*-value
	Low(n=30)	High(n=25)	
**Age**			0.765
young (< 60 years)	12	11	
old (⩾60 years)	18	14	
**Gender**			0.8
Male	19	15	
Female	11	10	0.491
**Stage**			
Ⅲ	16	11	
Ⅳ	14	14	
**Tumor differentiation**			0.96
Well+moderate	17	14	
poor	13	11	
**Tumor response**			0.04 *
CR+PR	20	10	
SD+PD	10	15	

* p<0.05

Supplement Table 3. Primer Sequences

mRNA	Primers
IL-11(human)	Forward: 5'-GTTGAGGAACTGATGGAGGACA-3'
	Reverse: 5'-TTGCACACATACACCAGGCTGT-3'
IL-11Rα	Forward: 5'-ACTTCCTGCTCAAGTTCCGT-3'
	Reverse: 5'-GGCACTGACTCGTACAGCAT-3'
GAPDH	Forward: 5'-GCACCGTCAAGGCTGAGAAC-3'
	Reverse: 5'-TGGTGAAGACGCCAGTGGA-3'

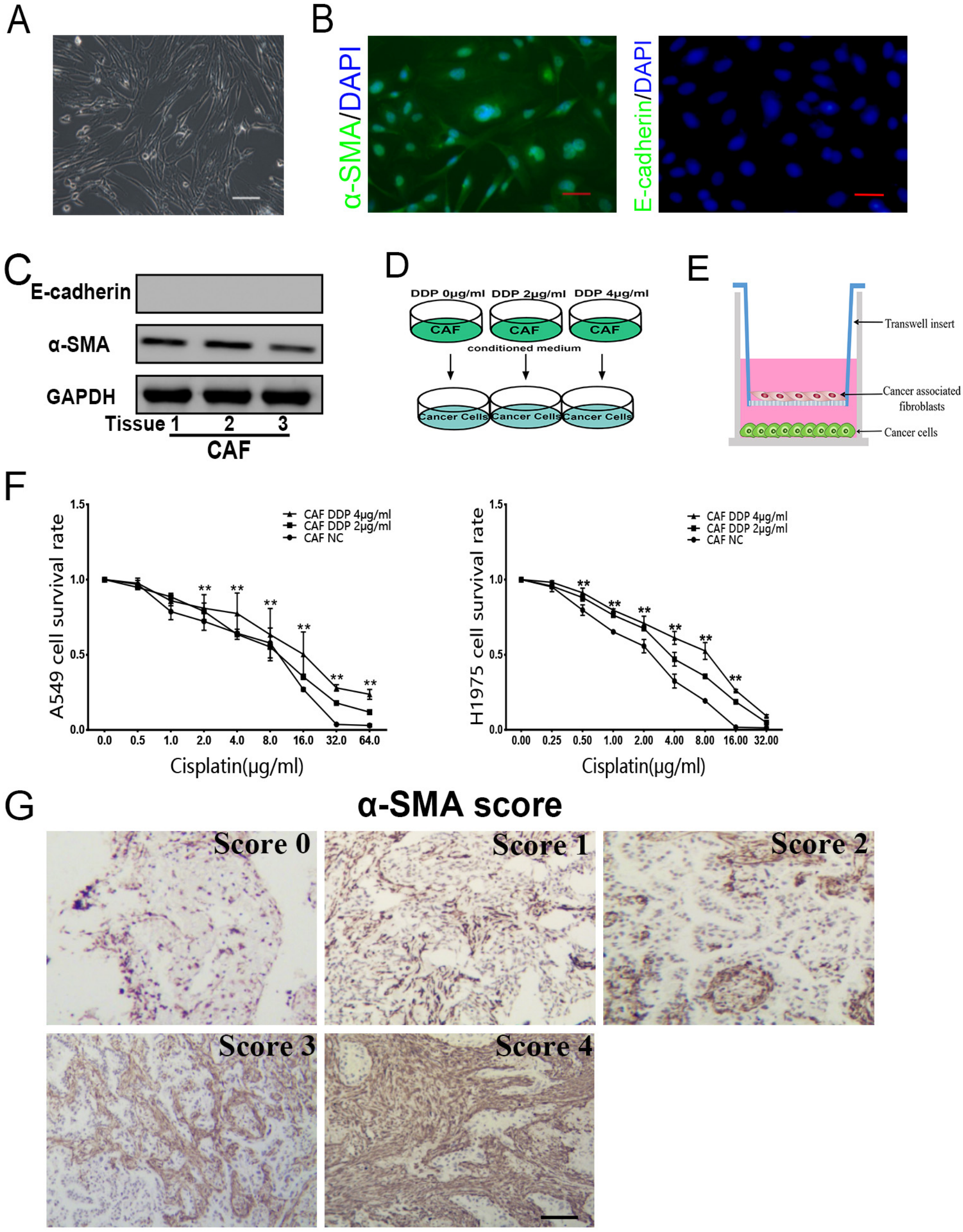
A
B
α-SMA/DAPI
E-cadherin/DAPI
C
E-cadherin
α-SMA
GAPDH
Tissue 1 2 3
CAF
D
DDP 0μg/ml
DDP 2μg/ml
DDP 4μg/ml
CAF
conditioned medium
Cancer Cells
E
Transwell insert
Cancer associated fibroblasts
Cancer cells
F
CAF DDP 4μg/ml
CAF DDP 2μg/ml
CAF NC
A549 cell survival rate
Cisplatin(μg/ml)
H1975 cell survival rate
Cisplatin(μg/ml)
G
α-SMA score
Score 0
Score 1
Score 2
Score 3
Score 4

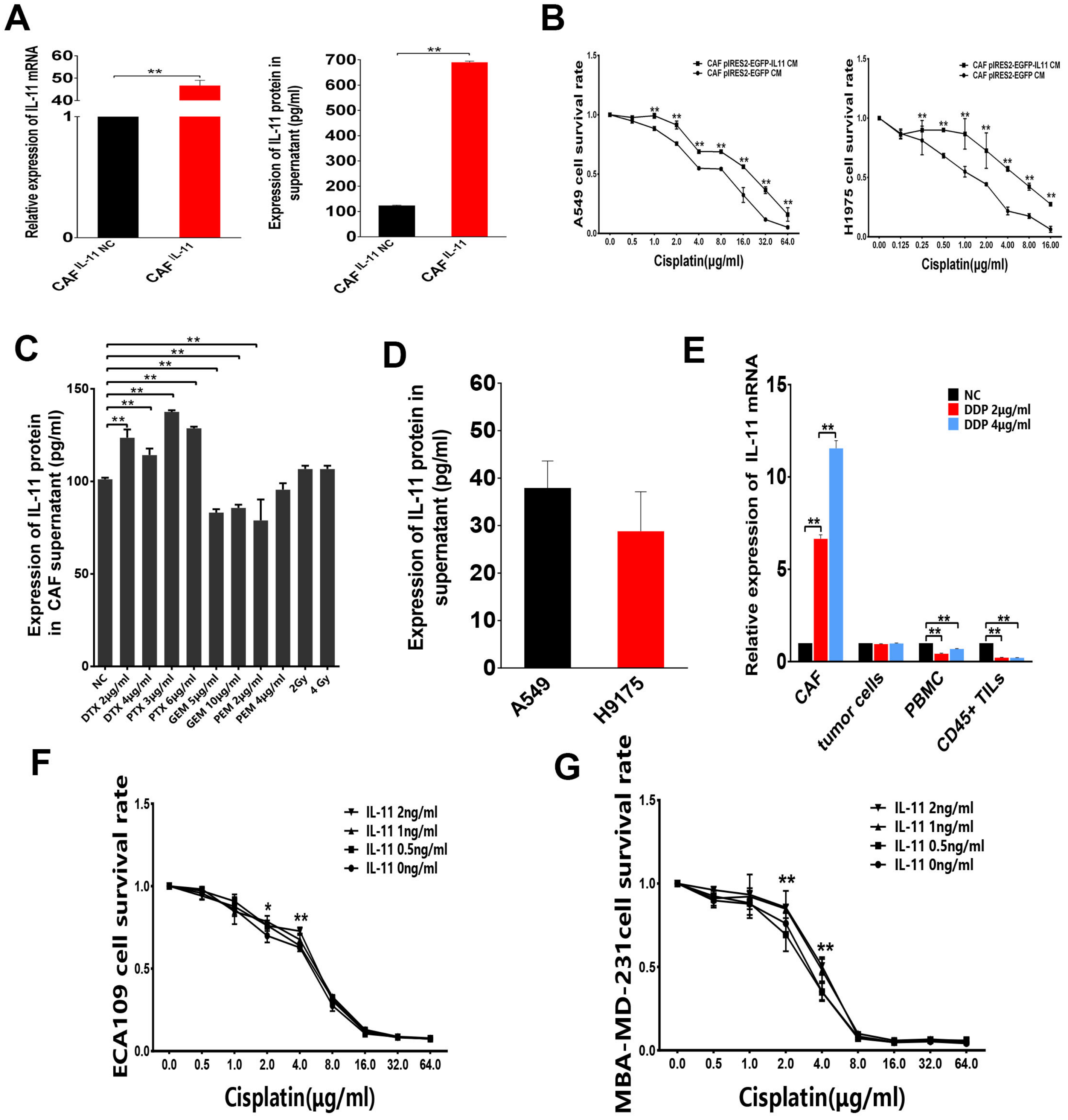

A
Relative expression of IL-11 mRNA
Expression of IL-11 protein in supernatant (pg/ml)
CAF IL-11 NC
CAF IL-11
B
A549 cell survival rate
H1975 cell survival rate
CAF pIRES2-EGFP-IL11 CM
CAF pIRES2-EGFP CM
Cisplatin(μg/ml)
C
Expression of IL-11 protein in CAF supernatant (pg/ml)
NC
DTX 2μg/ml
DTX 4μg/ml
PTX 3μg/ml
PTX 6μg/ml
GEM 5μg/ml
GEM 10μg/ml
PEM 2μg/ml
PEM 4μg/ml
2Gy
4 Gy
D
Expression of IL-11 protein in supernatant (pg/ml)
A549
H9175
E
Relative expression of IL-11 mRNA
NC
DDP 2μg/ml
DDP 4μg/ml
CAF
tumor cells
PBMC
CD45+ TILs
F
ECA109 cell survival rate
IL-11 2ng/ml
IL-11 1ng/ml
IL-11 0.5ng/ml
IL-11 0ng/ml
Cisplatin(μg/ml)
G
MBA-MD-231cell survival rate
IL-11 2ng/ml
IL-11 1ng/ml
IL-11 0.5ng/ml
IL-11 0ng/ml
Cisplatin(μg/ml)

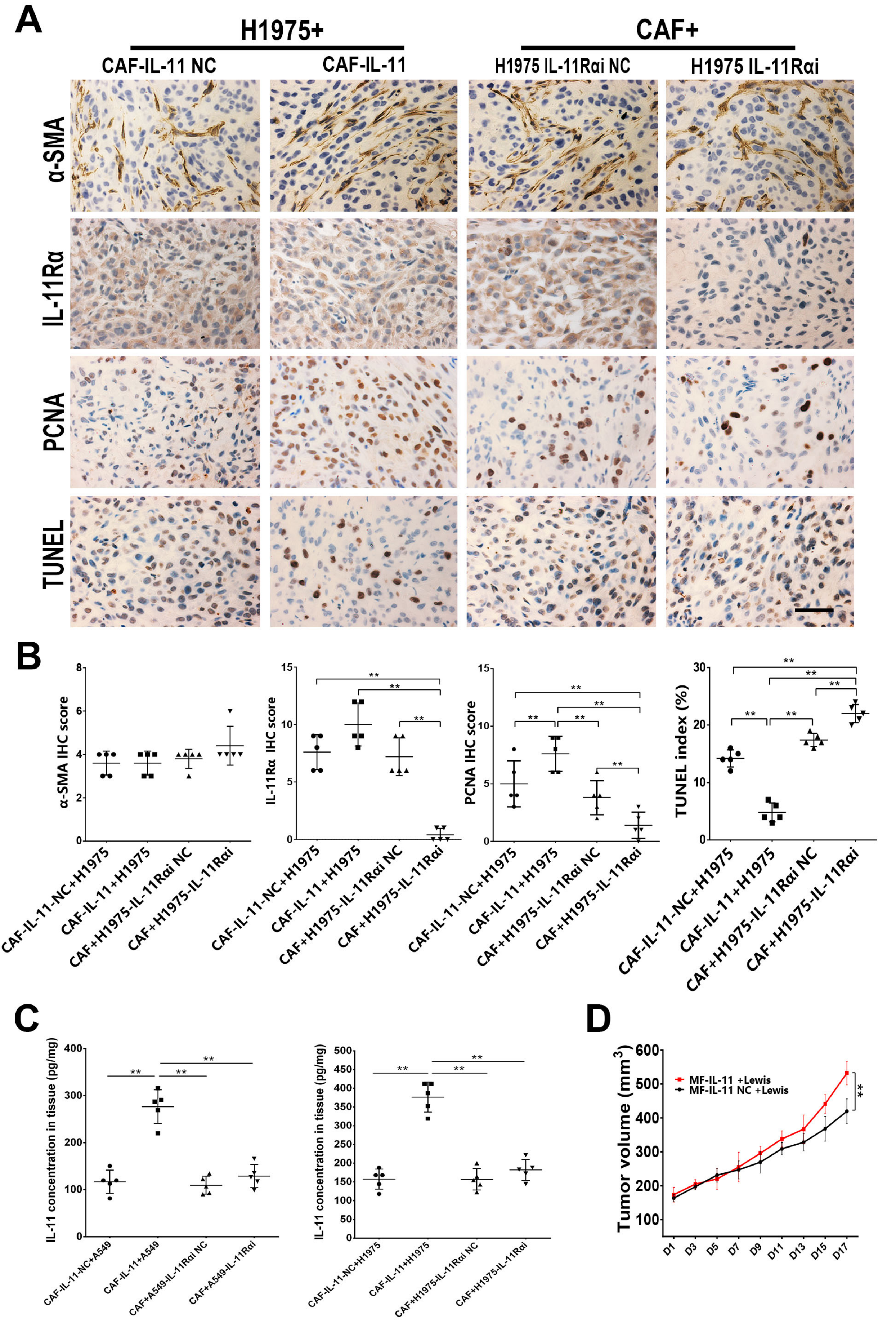
A
H1975+
CAF+
CAF-IL-11 NC
CAF-IL-11
H1975 IL-11Rαi NC
H1975 IL-11Rαi
α-SMA
IL-11Rα
PCNA
TUNEL
B
α-SMA IHC score
IL-11Rα IHC score
PCNA IHC score
TUNEL index (%)
CAF-IL-11-NC+H1975
CAF-IL-11+H1975
CAF+H1975-IL-11Rαi NC
CAF+H1975-IL-11Rαi
C
IL-11 concentration in tissue (pg/mg)
CAF-IL-11-NC+A549
CAF-IL-11+A549
CAF+A549-IL-11Rαi NC
CAF+A549-IL-11Rαi
D
Tumor volume (mm³)
MF-IL-11 +Lewis
MF-IL-11 NC +Lewis
D1
D3
D5
D7
D9
D11
D13
D15
D17

Figure 5C

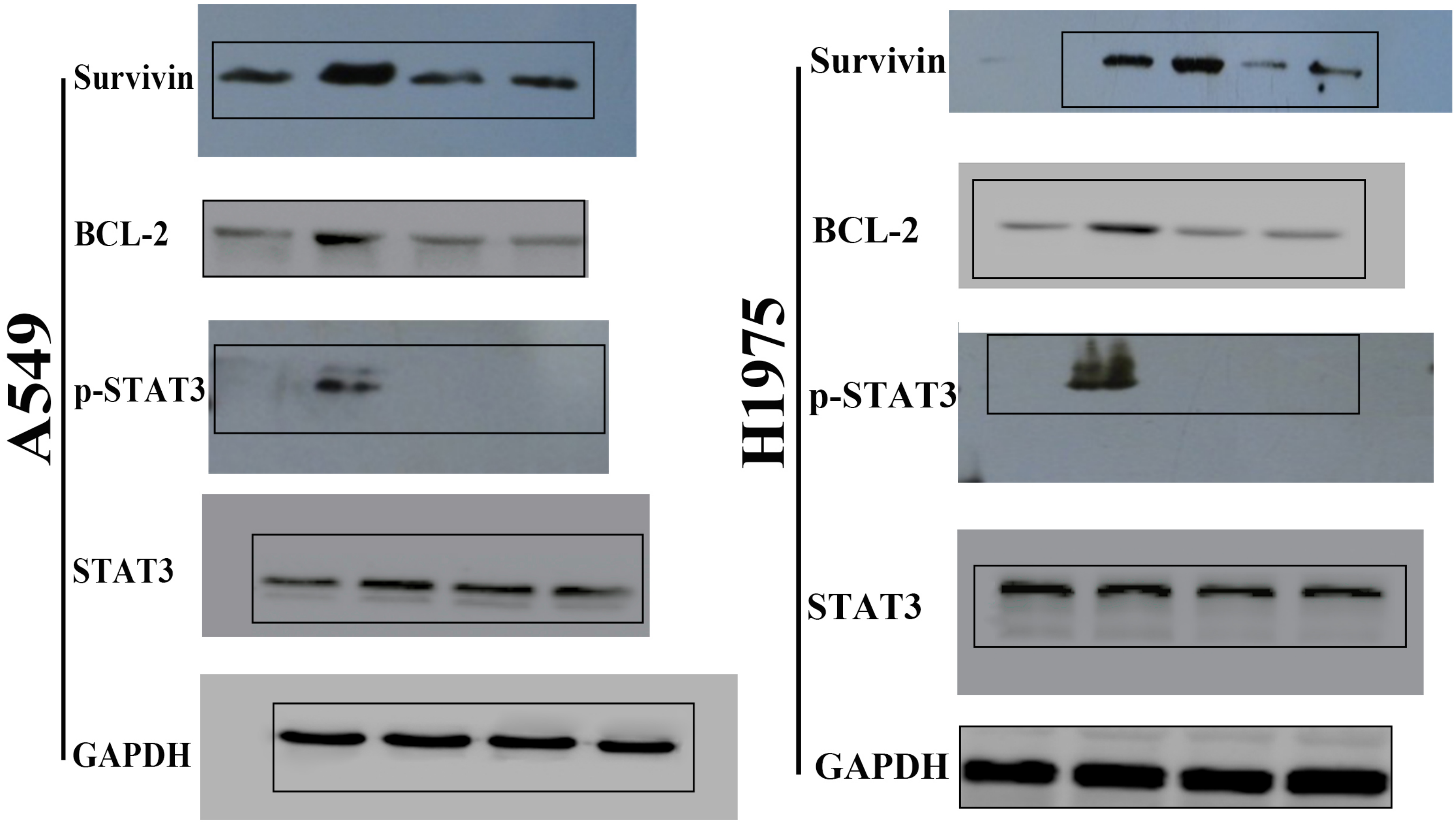

Figure S1C

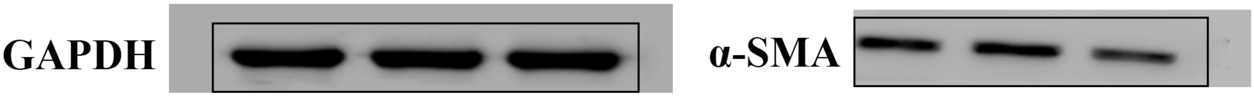

Supplement: Supplementary Files [file srep38408-s1.pdf]
